# Supplementary material for: Modelling Skylarks (Alauda arvensis) to Predict Impacts of Changes in Land Management and Policy: Development and Testing of an Agent-Based Model
Source: PLoS One. 2013 Jun 6;8(6):e65803. doi: 10.1371/journal.pone.0065803 (PMC3675089; doi:10.1371/journal.pone.0065803)
Supplement: Supporting Information S4 — The skylark ODdox as a zipped archive. (ZIP) [file pone.0065803.s004.zip › Skylark_ODdox/class_skylark___nestling-members.html]

ALMaSS Skylark ODdox: Member List


ALMaSS Skylark ODdox
 2.0


- Main Page
- Related Pages
- Classes
- Files

- Class List
- Class Index
- Class Hierarchy
- Class Members

Skylark\_Nestling Member List

This is the complete list of members for Skylark\_Nestling, including all inherited members.

|  |  |  |
| --- | --- | --- |
| AddStriglingMort(int lifestage) | Skylark\_Base | inline |
| Age | Skylark\_Base |  |
| BeginStep(void) | Skylark\_Nestling | virtual |
| CheckManagement(void) | TAnimal |  |
| CheckManagementXY(int x, int y) | TAnimal |  |
| CopyMyself() | TAnimal | inlinevirtual |
| CorrectWrapRound(void) | TAnimal | inlineprotected |
| CurrentStateNo | TALMaSSObject |  |
| DailyMortality(int mort) | Skylark\_Base | protected |
| Dying() | TAnimal | inlinevirtual |
| EndStep(void) | Skylark\_Nestling | virtual |
| InSquare(rectangle R) | Skylark\_Base |  |
| KillThis() | TAnimal | inlinevirtual |
| m\_Born\_x | Skylark\_Base |  |
| m\_Born\_y | Skylark\_Base |  |
| m\_CurrentSkState | Skylark\_Base |  |
| m\_Dad | Skylark\_Nestling | protected |
| m\_EM | Skylark\_Nestling | protected |
| m\_EM\_fail | Skylark\_Nestling | protected |
| m\_GrNeed | Skylark\_Nestling | protected |
| m\_Location\_x | TAnimal | protected |
| m\_Location\_y | TAnimal | protected |
| m\_MyHome | Skylark\_Base |  |
| m\_NestLeavingChance | Skylark\_Nestling | protected |
| m\_OurLandscape | TAnimal | protected |
| m\_OurPopulationManager | Skylark\_Base |  |
| m\_OurTerritories | Skylark\_Base |  |
| m\_pcide\_conc | Skylark\_Base |  |
| m\_pesticide\_accumulation | Skylark\_Base |  |
| m\_Size | Skylark\_Base |  |
| On\_FoodSupply(double food) | Skylark\_Nestling | virtual |
| OnArrayBoundsError() | TALMaSSObject |  |
| OnDadDead() | Skylark\_Nestling |  |
| OnDeserted() | Skylark\_Nestling |  |
| OnFarmEvent(FarmToDo event) | Skylark\_Nestling | protectedvirtual |
| OnYouHaveBeenEaten() | Skylark\_Nestling |  |
| PesticideResponse(void) | Skylark\_Nestling | protectedvirtual |
| SetX(int a\_x) | TAnimal | inline |
| SetY(int a\_y) | TAnimal | inline |
| Sex | Skylark\_Nestling | protected |
| Skylark\_Base(int x, int y, SkTerritories \*Terrs, Landscape \*L, Skylark\_Population\_Manager \*SPM, int bx, int by, int mh) | Skylark\_Base |  |
| Skylark\_Nestling(int x, int y, Skylark\_Male \*Daddy, Landscape \*L, SkTerritories \*Terrs, Skylark\_Population\_Manager \*SPM, int bx, int by, int mh) | Skylark\_Nestling |  |
| st\_Developing() | Skylark\_Nestling | protectedvirtual |
| st\_Dying() | Skylark\_Nestling | protectedvirtual |
| st\_Maturing() | Skylark\_Nestling | protectedvirtual |
| Step(void) | Skylark\_Nestling | virtual |
| StepDone | TALMaSSObject |  |
| Supply\_m\_Location\_x() | TAnimal | inline |
| Supply\_m\_Location\_y() | TAnimal | inline |
| SupplyFarmOwnerRef() | TAnimal |  |
| SupplyPolygonRef() | TAnimal | inline |
| SupplyPosition() | TAnimal |  |
| TALMaSSObject() | TALMaSSObject |  |
| TAnimal(int x, int y, Landscape \*L) | TAnimal |  |
| WhatState() | Skylark\_Base | inlinevirtual |
| ~TALMaSSObject() | TALMaSSObject | virtual |


- Generated on Thu Jan 10 2013 13:15:38 for ALMaSS Skylark ODdox by
   1.8.1.1
